# Challenges and research opportunities for lung cancer screening in China

**DOI:** 10.1186/s40880-018-0305-0

**Published:** 2018-06-07

**Authors:** Zixing Wang, Yuyan Wang, Yao Huang, Fang Xue, Wei Han, Yaoda Hu, Lei Wang, Wei Song, Jingmei Jiang

**Affiliations:** 1 Department of Epidemiology & Biostatistics, Institute of Basic Medical Sciences, Chinese Academy of Medical Sciences, Beijing, 100005 P. R. China; 2 Radiology Department, Cancer Hospital, Chinese Academy of Medical Sciences, Beijing, 100021 P. R. China; 3 Radiology Department, Peking Union Medical College Hospital, Chinese Academy of Medical Sciences, Beijing, 100730 P. R. China

**Keywords:** Lung cancer, Low-dose CT, Screening

## Abstract

Following publication of the results of the National Lung Screening Trial in the United States, a randomized controlled trial in Italy (ITALUNG) and two simulation studies in China reported similar findings in 2017 favoring lung cancer screening with low-dose computed tomography among smokers. With such advances in lung cancer screening, worldwide interest has gradually shifted from evaluating whether refining lung cancer screening protocols is effective in preventing deaths. However, there are several practical problems to be resolved, including the balance of enrollment criteria and cost effectiveness, precise measurements to reduce false positive findings, risk-based optimization of screening frequency, challenges associated with cancer heterogeneity, strategies to combine image screening with novel biomarkers, dynamic monitoring of the natural history of cancer, accurate identification and diagnosis of cases among huge populations, and the impact of tobacco control policy and environment protection. As one in three individuals with lung cancer worldwide resides in China, these questions pose great challenges as well as research opportunities for population screening programs in China.

One in five deaths from lung cancer can be averted by conducting low-dose computed tomography (LDCT) screening among smokers, as evidenced by results from the United States (US) National Lung Screening Trial (NLST) in 2011 [1]. However, the NLST is the only available randomized trial with the statistical strength to prove such a conclusion. In April 2017, a smaller-scale Italian trial (ITALUNG) involving four rounds of annual screening and a maximum follow-up of 10 years published its final results [2]. ITALUNG randomly assigned 1613 smokers to a LDCT screening group and 1593 smokers to a usual care group. Although statistical significance was not achieved because of the small sample size, the results showed a 17% reduction in overall mortality and a 30% reduction in lung cancer-specific mortality with LDCT. Two simulation studies that favored LDCT screening in China were also published this year, and similar conclusions were reached, although different models and parameters were used [3, 4].

With a micro-simulation model adapted from the US National Cancer Institute, Sheehan et al. [3] predicted the effects of lung cancer screening with LDCT in China between 2016 and 2050 and published their results in March 2017. Screening effects were compared between screening eligibility criteria defined in the 2015 Chinese lung cancer screening guidelines [5] as well as in the US version [6]. Model input parameters included smoking and cessation rates and mortality extracted from global reports for various causes including lung cancer. By assuming 100% adherence, the authors estimated that screening with the two eligibility criteria would prevent 0.7 million deaths in the 35-year timeframe. They estimated population-level reductions in mortality of 6.30% in males and 2.79% in females with the Chinese criteria and 6.58% in males and 1.97% in females with the US criteria. Comparison of their results with the NLST showed that among a specified screened cohort (1960s), 15.1% and 19.7% reductions in mortality were achieved using LDCT screening compared with no screening according to Chinese and US eligibility criteria, respectively.

Another study published in July 2017 by Wang et al. [4] analyzed lung cancer mortality differences in urban China with LDCT screening, chest X-ray screening, and without screening. A decision tree model was used, with considerations of special screening issues such as “missed diagnosis,” “false positive diagnosis,” and “over diagnosis”. Model inputs were primarily based on Chinese studies or national registry data and included prevalence of lung cancer, LDCT and X-ray sensitivities and specificities, and stage-specific survival rates. In terms of eligibility criteria, screening was assumed among urban smokers aged 45–80 years in a base case scenario. The results showed that among such a screened population, with LDCT screening led to 17.2% mortality reduction compared with chest X-ray screening and 24.2% mortality reduction compared with no screening. If more rigorous age criteria were used, screening efficiency with LDCT may be slightly improved, for example, to 17.4% mortality reduction compared with chest X-ray screening and 24.3% mortality reduction compared with no screening if the 2015 Chinese guideline criteria are used.

Generally, the results from ITALUNG and the two simulation studies in China were similar and consistent with the previous NLST report. This strengthens the feasibility of LDCT screening for lung cancer. In addition, the Dutch-Belgian lung cancer screening trial (NELSON), currently the largest ongoing lung cancer screening trial, is nearing the reporting of mortality outcomes [7]. If similar favorable results are released, a wide-ranging boom in lung cancer screening similar to that following publication of the NLST results could be expected.

The results of these two simulation studies are timely and meaningful, as one in three individuals with lung cancer resides in China [8]. In fact, there are several ongoing large-scale lung cancer screening programs among the Chinese population [9]. However, recent advances revealed a number of practical problems that are yet to be resolved. As studies on cancer screening generally call for a large number of subjects and a long follow-up period, the new challenges for lung cancer screening discussed below also provide unique research opportunities for China, especially considering that the majority of large-scale trials in the US and Europe are completed or nearing completion [10].

## Study area 1: quantitative analysis-based population screening strategy

### 1. Enrollment criteria for screening and cost effectiveness

A major conclusion of the two recent studies [3, 4] on lung cancer screening in China was the emphasis on deliberately selecting candidates for screening. According to a study published by Katki et al. [11] in 2016, several screening effectiveness and efficiency metrics (e.g., number needed to screen to prevent one lung cancer death) could be optimized in the US with a “risk-based approach” for selecting the high-risk population. This is especially important for China, where the trade-off between effectiveness and costs should be considered for most resource-limited areas. On the other hand, when we consider feasibility of screening in an extended population living with favorable conditions, there is currently no evidence that younger non-smoking patients with lung cancer could not benefit. For example, long-term screening experience in Hitachi district in Japan showed that prognoses of non/light smokers with lung cancer detected in screening were favorable, which provided the rationale for a trial investigating screening among non/light smokers in Japan [12]. Similarly, the results from the Cancer Hospital Chinese Academy of Medical Sciences also indicated the necessity for screening among women who were passive smokers with a minimum age of 40 years [13]. Therefore, more work, including statistical modeling, needs to be done to refine region-specific screening eligibility criteria and to balance effectiveness and cost.

### 2. Reducing false-positive findings on LDCT images

According to Wang et al.’s work [4], effectiveness of lung cancer screening should be further improved by solving some bottleneck technical problems with the aim to optimize population outcomes, the difficulty in differentiating early stage cancers from benign nodules being the most influential problem. The high false-positive rate of LDCT images resulted in a considerable percentage (24.2%) of screened participants requiring further examination, 96.4% of whom were not finally diagnosed with lung cancer in the NLST [12]. As revealed in Wang et al.’s study [4], false-positive findings may lead to diminished or negative gains in quality of life due to anxiety experienced before final diagnosis and are also associated with a 3.5-fold risk of death due to unnecessary diagnosis and treatment. Although the volume doubling time showed strong potential for determining malignancy in NELSON [14], volumetric analysis is not applicable to non-solid nodules and is heavily reliant on subsequent screening. Therefore, for a better and timely judgment about benign and malignant nodules, more quantitative analysis approaches and empirical validation studies are needed.

### 3. Determining screening frequency and optimization of intervals

The NELSON trial used three intervals between screens (1, 2, and 2.5 years) to determine the best screening frequency. A recent report from that study observed a significantly increased rate of “missed” cancers and a decreased proportion of early-stage cancers with the longest screening interval [15], which highlights the debate between annual and biennial screening, as discussed in other studies [16]. Compared with fixed intervals, a better choice may be adjusting screening intervals according to individual risk of lung cancer. However, no evidence is available regarding this option, and a more elaborate protocol for determining and changing individual screening frequencies is needed. In contrast to developing models for the selection of screening candidates, models to calculate individual risk for the purpose of optimizing screening intervals can be based on both patient characteristics and lesion signs detected in previous LDCT images [17]. Such comprehensive modeling with multivariate and heterogeneous data would certainly contribute to this but presents more challenges.

## Study area 2: precise screening with insights from molecular studies

### 4. Understanding cancer heterogeneity and its impact on screening

The high heterogeneity of cancer is another issue that has important implications for lung cancer screening. According to our recent meta-analysis [18], indolent cancers contributed to over-diagnosis, as they comprised a large proportion of cancers additionally detected with LDCT. In addition, no evidence of superiority with LDCT for the early detection of small cell lung cancers was observed, unlike non-small cell lung cancers. Therefore, more pathologic type-specific analyses with a sufficient number of cases are needed to quantify the degree to which “early detection” translates into real “increased lives.” With the recent surge in precision medicine studies on molecular reconstruction of cancer classification systems [19], bridging such studies with lung cancer screening may present new opportunities and provide more enlightening insights.

### 5. Combination of LDCT with novel biomarkers

Although evidence from population-level studies is scarce, biomarkers are considered the best way to assist in lung cancer screening [20]. They can be used in various stages of screening, including selecting high risk populations, serial screening tests, and establishing molecular diagnoses for further individualized clinical management. However, challenges remain regarding the discovery, optimal selection and validation of markers. Research frameworks and standards (including statistical requirements) to routinize the whole process are lacking; subject numbers are usually small because of a disconnection between basic research and clinical resources; and failures are common in replicating the claimed diagnostic value when applied to patients in different cancer stages or with greater tumor heterogeneity [21]. As discussed earlier, integrating molecular studies into LDCT screening programs provides a unique opportunity to resolve some of these problems. This modality has already been adopted in some recent large programs [22–24]. For example, a biomarker panel composed of plasma DNA and genomic analyses in the ITALUNG biomarker study was as sensitive as LDCT (90%) and increased screening specificity from 71% (LDCT alone) to 89% via a multimodal approach [22]. Another diagnostic test consisting of seven autoantibodies is currently under a randomized controlled trial among 12,000 Scotsmen, aiming to examine whether pre-screening with this test would be helpful to identify high-risk candidates, thereby increasing the overall effectiveness of LDCT screening [23].

### 6. Dynamic monitoring of cancer natural history

An inherent but usually neglected advantage of lung cancer screening studies is the ability to gain a better understanding of the natural history of the disease. A recent report from the NELSON trial revealed that the volume growth pattern of screen-detected lung cancers can be fitted to an exponential function [25]. In a prospective cohort study, Jamal-Hanjani et al. [26] tracked the evolution of non-small cell lung cancer and observed detailed data about clonal and sub-clonal events using multiregion whole-exome sequencing. These reports indicated the start of an era of dynamic lung cancer studies from both morphological and molecular perspectives. More refined protocols for lung cancer screening may be obtained when we have a clearer picture of the macroscopic and microscopic evolutional processes.

## Study area 3: dealing with more practical challenges in the real world

### 7. Integration of databases and assurance of accurate cases

As incidences and mortality of cancers are relatively low, a longitudinal cancer study calls for a large number of subjects and is heavily reliant on the accuracy of collecting and confirming incident or mortal cases. This task is challenging for lung cancer screening studies, particularly when participants were not diagnosed at the hospital that conducted the screening. A project was recently initiated in Shandong Province, China, that aimed to create a database linking the cancer registry with health insurance claims [27], which suggests a way to facilitate such large-scale population-based studies. However, as noted by the NELSON study team [28], registry data are not 100% sensitive and accurate. Therefore, more mechanisms need to be developed to guarantee accuracy of these primary outcomes.

### 8. Influences of tobacco control policy and environment protection

Smoking and other environmental factors are well-known risk factors that are targeted in primordial or primary prevention of lung cancer. As screening is a secondary prevention approach, secular variation in these risk factors may have non-linear effects on lung cancer incidence [29] and, thus, may complicate the predicted effectiveness of screening. In addition, cancer screening motivated smokers to quit, as ex-smokers concerned with their health status were more willing to participate in the United Kingdom Lung Cancer Screening Trial [30]. Therefore, socio-behaviors should be carefully monitored and considered to take advantage of combining programs such as tobacco control and lung cancer screening. A third important factor is that the spectrum of cancer pathological type may change along with changes in environmental risk factors, which further complicates the issue. Therefore, future research should also be placed in a broader context and investigate the impact of policies on tobacco control and environment protection, which are currently undergoing significant changes in China.

In summary, research on lung cancer screening is about to reach a turning point that will shift the focus from evaluating whether the refinement of such screening protocols for increased cost efficiency is effective in reducing deaths. Several challenges and major research opportunities remain to be considered in future studies, especially studies conducted in China where more than 0.5 million people may benefit from such scientific progress.

## Abbreviations

LDCT: low-dose computed tomography
NLST: National Lung Screening Trial
US: United States
NELSON: Dutch-Belgian lung cancer screening trial (Dutch language)
